# Inferring Phenotypic Properties from Single-Cell Characteristics

**DOI:** 10.1371/journal.pone.0037038

**Published:** 2012-05-25

**Authors:** Peng Qiu

**Affiliations:** Department of Bioinformatics and Computational Biology, The University of Texas M. D. Anderson Cancer Center, Houston, Texas, United States of America; Mount Sinai School of Medicine, United States of America

## Abstract

Flow cytometry provides multi-dimensional data at the single-cell level. Such data contain information about the cellular heterogeneity of bulk samples, making it possible to correlate single-cell features with phenotypic properties of bulk tissues. Predicting phenotypes from single-cell measurements is a difficult challenge that has not been extensively studied. The 6th Dialogue for Reverse Engineering Assessments and Methods (DREAM6) invited the research community to develop solutions to a computational challenge: classifying acute myeloid leukemia (AML) positive patients and healthy donors using flow cytometry data. DREAM6 provided flow cytometry data for 359 normal and AML samples, and the class labels for half of the samples. Researchers were asked to predict the class labels of the remaining half. This paper describes one solution that was constructed by combining three algorithms: spanning-tree progression analysis of density-normalized events (SPADE), earth mover’s distance, and a nearest-neighbor classifier called Relief. This solution was among the top-performing methods that achieved 100% prediction accuracy.

## Introduction

Flow cytometry technology provides multi-parametric single-cell measurements of a heterogeneous population of cells [1]. The flow cytometry data for one biological sample is usually in the form of a tall thin matrix, where each row corresponds to an individual cell and each column corresponds to one protein marker. Each element in the data matrix is the expression of a protein marker on/inside an individual cell. Such single-cell data contain information about the cellular heterogeneity underlying the measured population (i.e., how many cell types there are, and the percentages of cells belonging to each cell type). If such data for multiple samples are available, the relationship between the cellular heterogeneity and the phenotypic properties of the samples can be evaluated.

The relationship between single-cell characteristics and phenotypic properties has been discussed in the literature. For example, flow cytometry was used to derive the percentages of smudge cells and lymphocytes in blood samples, which were shown to be predictive of prognosis for patients with chronic lymphocytic leukemia [2]. CD33 expression derived from flow cytometry predicted clinical outcome in patients with acute myeloid leukemia (AML) who were treated with gemtuzumab ozogamicin monotherapy [3]. Flow cytometry was used to profile follicular lymphoma tumors and identify a subpopulation of lymphoma cells with impaired B-cell antigen receptor signaling, whose abundance was negatively correlated with survival [4]. These studies demonstrate possible correlations between cellular compositions and clinical outcomes, such as survival and drug response.

To correlate single-cell data and phenotypic properties, in general, two computational components are needed: (1) identify cell types or subpopulations of cells, and (2) infer phenotypic properties from summary statistics of the subpopulations. For subpopulation identification, the most widely used approach for analyzing flow cytometry data is gating, which is a subjective and labor-intensive method that relies on user-defined sequences of nested biaxial plots [5], [6]. To reduce the subjectivity, a number of automated clustering algorithms have been proposed, such as K-means [7], [8], mixture models, [9]–[12], density-based clustering [13], [14], spectral analysis [15], and tree-based analysis [16], [17]. Once the subpopulations are defined, summary statistics can be easily computed (i.e., percentages and median marker expressions). For the purpose of inferring phenotypic properties, many classification algorithms in the machine learning literature can be applied. Examples include support vector machine [18], neural network [19], random forest [20], model-based classifiers [21], and nearest neighbor approaches [22], [23]. The combination of subpopulation identification and classification algorithms can produce many analysis pipelines, each of which may have its own strengths and weaknesses.

The challenges put forth by DREAM, the acronym for Dialogue for Reverse Engineering Assessments and Methods, provide objective and unbiased platforms for evaluating computational methods in systems biology [24]–[27]. Started in 2007, the DREAM project designs a set of computational challenges each year, invites scientists to solve them, and evaluates the solutions that are submitted. The challenges have included: transcription factor binding prediction, network inference, missing data prediction, sequence motif recognition, parameter estimation, and gene expression prediction. The DREAM6 in 2011 included one challenge on AML prediction using single-cell flow cytometry data. Operating in parallel to DREAM, another initiative FlowCAP (Flow Cytometry: Critical Assessment of Population Identification Methods) focuses on computational methods for flow cytometry analysis. The AML prediction challenge was shared by DREAM6 and FlowCAP2.

This manuscript discusses my participation in the AML prediction challenge. The challenge provided flow cytometry data for 359 subjects and the normal/AML status of 179 subjects. Researchers were asked to predict the normal/AML status of the remaining 180 subjects. In response to this challenge, I submitted one solution and it achieved 100% prediction accuracy. The main components of the solution were: spanning-tree progression analysis of density-normalized events (SPADE) [16], earth mover’s distance (EMD) [28] and a nearest-neighbor classification approach called Relief [22]. Detailed descriptions of the challenge and my solution are provided hereafter.

## Results

### Description of the AML Prediction Challenge and Data

The AML prediction challenge included a total of 359 samples from 316 healthy donors and 43 AML positive patients. Each sample was subdivided into 8 aliquots/tubes, stained for different marker combinations, and assayed by flow cytometry. Tube 1 was an isotype control and tube 8 was unstained. Table 1 shows the marker combinations, five protein markers per tube. In addition to the protein markers, the data contained measurements for forward scatter (FSC) and side scatter (SSC) for each cell, which reflected cell size and granularity. Therefore, the data of each tube was a matrix containing 7 columns. The total number of cells (rows) varied across the different tubes and samples, ranging from 8000 to 50000.

**Table 1:** The fluorophore-conjugated antibodies contained in each of the 8 tubes.

	FL1	FL2	FL3	FL4	FL5
Tube 1	IgG1-FITC	IgG1-PE	CD45-ECD	IgG1-PC5	IgG1-PC7
Tube 2	Kappa-FITC	Lambda-PE	CD45-ECD	CD19-PC5	CD20-PC7
Tube 3	CD7-FITC	CD4-PE	CD45-ECD	CD8-PC5	CD2-PC7
Tube 4	CD15-FITC	CD13-PE	CD45-ECD	CD16-PC5	CD56-PC7
Tube 5	CD14-FITC	CD11c-PE	CD45-ECD	CD64-PC5	CD33-PC7
Tube 6	HLA-DR-FITC	CD117-PE	CD45-ECD	CD34-PC5	CD38-PC7
Tube 7	CD5-FITC	CD19-PE	CD45-ECD	CD3-PC5	CD10-PC7
Tube 8	NonSpecific	NonSpecific	NonSpecific	NonSpecific	NonSpecific

The information for 179 samples regarding whether they were healthy or AML positive was provided as the training set. The challenge was to predict the normal/AML status of the remaining 180 testing samples. It is worth noting that since the total numbers of normal and AML samples were provided, participants were able to easily figure out the numbers of normal and AML samples in the testing samples, which were 160 and 20, respectively.

As shown in Table 1, only one protein marker was shared by different tubes. Since the number of overlapping markers in the different tubes was small (CD45, FSC and SSC), I decided to analyze the 8 tubes separately, as if there were 8 different prediction problems. In the following subsections, tube 2 will be used to illustrate the analysis and results, because it is the first tube that is not a control tube.

### Data Quality Check and Preprocessing

The flow cytometry data of each tube for each sample were provided in a comma-separated values (CSV) file. The total number of files was 359*8 = 2872. Data in the CSV files were compensated and transformed [29] before being released to the participants. FSC was transformed in linear scale, while SSC and the five protein markers were transformed in logarithmic scale.

**Figure 1:**
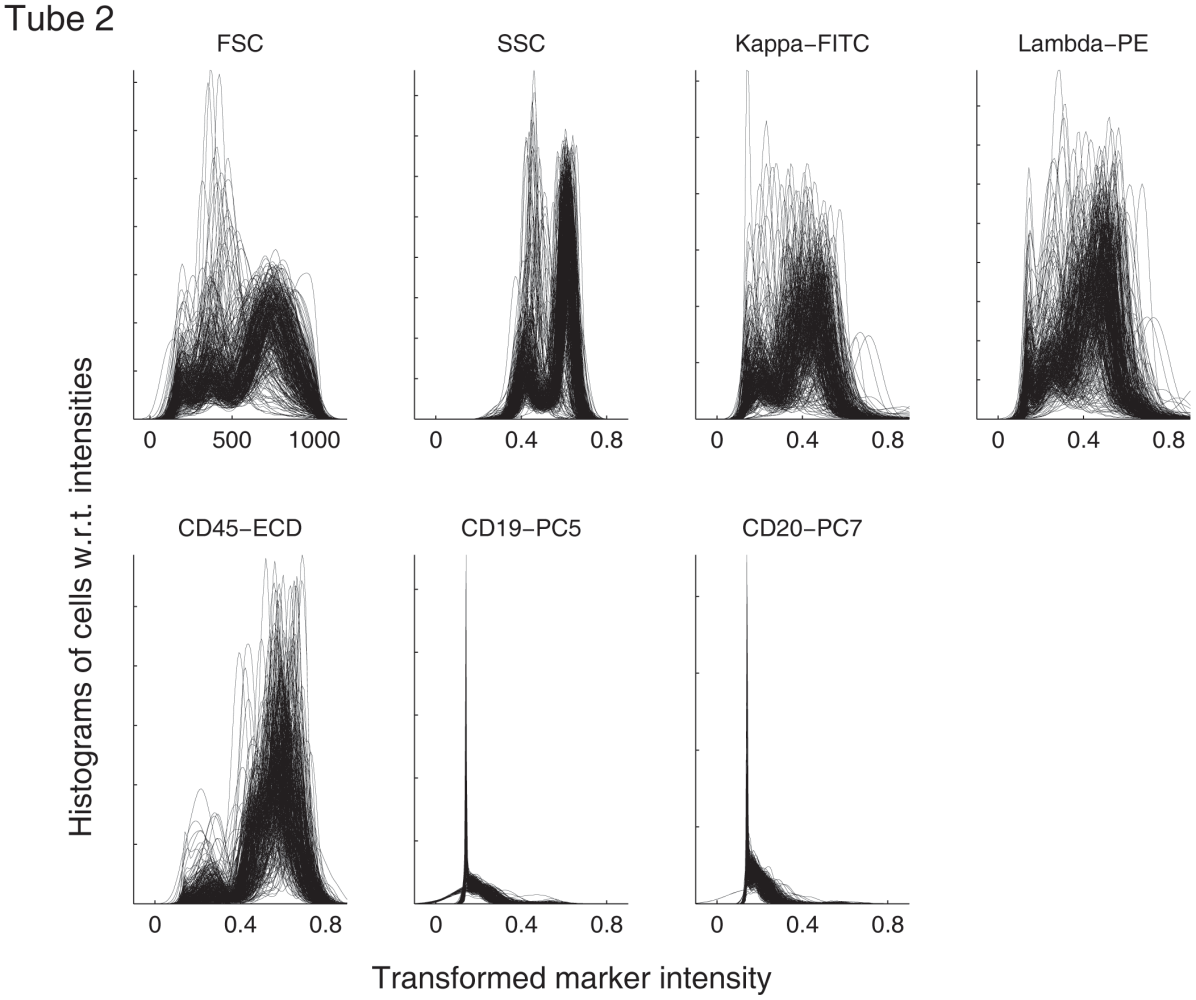
Distributions of marker intensities in data of tube 2. Each panel contains 359 curves, and each curve shows the distributions of the intensities of one marker in tube 2 for one of the 359 samples.

As a quality check, histograms were visualized for each marker in each file. For example, Figure 1 shows the histograms derived from tube 2. Each plot contains 359 curves, which are the distributions of the transformed intensities for one marker in the 2nd tube of the 359 samples. The curves in each plot formed clusters of peaks, meaning that the distributions of the intensities of the markers in tube 2 were relatively well aligned across different samples. Similar patterns were observed in the data from the other tubes (see information S1). Such observations indicated that there was no significant mean shift or variance change among the samples, and thus, data from different samples were directly comparable without the need for normalizing any baseline differences among samples.

From Figure 1, it can be observed that all markers in the logarithmic scale shared a similar standard deviation (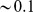); whereas the standard deviation of FSC was large, because of its linear scale. To ensure that the subsequent analysis was not dominated by the FSC channel, linear transformation was used to shift the mean of the FSC data to 0 and scale the standard deviation to 0.1. This was performed separately for each data file.

### SPADE

SPADE is the acronym for spanning-tree progression analysis of density-normalized events [16]. It is a computational approach for flow cytometry analysis. SPADE views a flow cytometry dataset as a point cloud of cells and derives a tree structure to represent the geometry of the cloud, which reflects the cellular heterogeneity underlying the data. To achieve this, SPADE contains four computational components: density-dependent downsampling, agglomerative clustering, minimum-spanning tree construction, and upsampling. As mentioned above, data for different tubes were analyzed separately. In this subsection, tube 2 is used to illustrate how SPADE was applied for the AML prediction challenge.

To jointly analyze the tube 2 data for all the samples with SPADE, my strategy was to first perform density-dependent downsampling on the tube 2 data for the individual samples separately, then pool the downsampled data for clustering and minimum-spanning tree construction, and finally apply upsampling to calculate the distribution of cells with respect to the tree for each sample.

Density-dependent downsampling is a process that removes cells. This process removes cells in the abundant cell types with high probability while keeping most cells in the rare cell types. Downsampling was performed on individual samples because the total numbers of cells in different samples were different. To ensure that individual samples contributed equally to the collective when the downsampled data for all samples were pooled, the downsampling parameters of SPADE were varied such that the same number of cells (i.e., 2000 in this analysis) survived the downsampling process for each sample.

**Figure 2:**
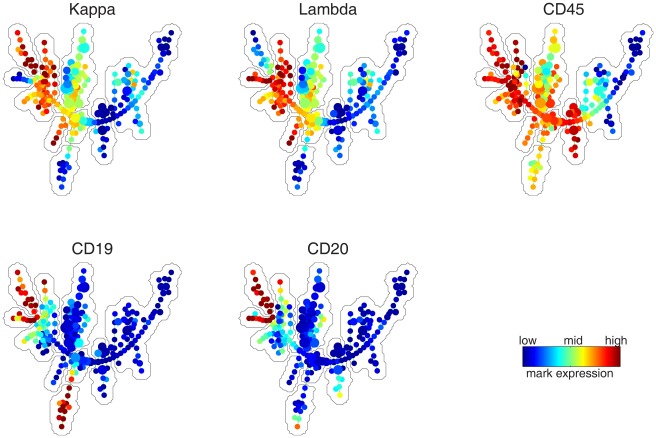
SPADE tree derived from tube 2 data of all 359 samples. Each tree is colored by the expression of one protein marker in tube 2: kappa, lambda, CD45, CD19 and CD20. Manually derived annotation boundaries are shown by the gray curves that partition the tree. These boundaries facilitate the interpretation of which phenotype is represented by different parts of the tree.

The downsampled data for all samples were pooled, forming a meta-cloud that represented the union of all phenotypes present in at least one sample. Agglomerative clustering was applied to divide the meta-cloud into small pieces (i.e. 150 clusters). Minimum-spanning tree construction was used to derive a tree structure that connected the clusters with minimum total edge length. Each tree node represented one cluster of cells that were similar to each other, which occupied one small region of the meta-cloud. The topology of the tree approximated the skeleton of the meta-cloud. Figure 2 shows multiple versions of the SPADE tree. The only difference among those versions is the coloring. The nodes of each tree were colored by the median intensity of one marker measured in tube 2.

The topology, layout and coloring of the tree were automatically generated by SPADE. Annotation boundaries in Figure 2 were manually drawn to partition the tree into subgraphs, such that the color pattern within one boundary was relatively homogeneous in all the colored trees. For example, nodes in the boundary that covered the upper-right branch were negative for all five protein markers; nodes within the adjacent annotation boundary was positive for CD45 and negative for the other four protein markers. Each annotation boundary outlined one branch/subgraph of the tree, which might correspond to a subpopulation of cells with a distinct phenotype. Since tube 2 measured B-cell markers, a few branches of the SPADE tree can be interpreted as B-cell subtypes. The two branches in the upper-left corner were mature B cells because they were positive for both CD19 and CD20. This was further confirmed by the mutually exclusive expression of kappa and lambda in those two branches, which has been observed in mature B cells [30]. The bottom branch was CD19+ CD20- Kappa- Lambda-, which was likely to be immature B cells. The five subgraphs near the center of the tree were CD45+ CD19- CD20-, with different expressions of kappa and lambda. The cell types of nodes in those subgraphs were not clear according to the markers in tube 2. The manually derived annotaion boundaries were useful for understanding the correspondence between the tree and the underlying cell types. However, for the purpose of predicting the normal/AML classification, such annotations were not necessary.

**Figure 3:**
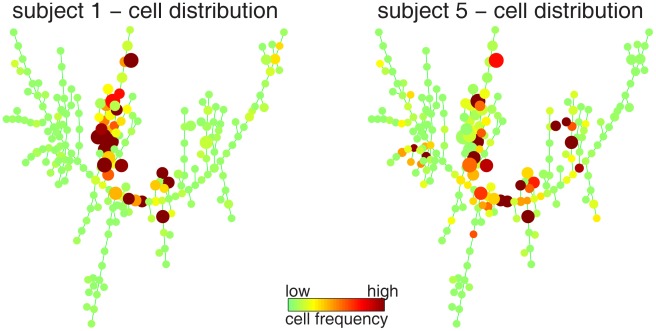
SPADE tree derived from tube 2, colored by the distribution of cells in two individual samples. Subject 1 is a healthy donor and subject 5 is a patient with AML.

After the SPADE tree was derived from the pooled downsampled data, upsampling was performed to map each cell in the original dataset to the node to which it was most similar. Through this process, for each sample, every cell in the original dataset was assigned to one tree node, which enabled the calculation of the percentage of cells that belonged to each tree node. The percentage values could also be used to color the SPADE tree. Figure 3 shows two examples. Each plot highlighted the parts of the meta-cloud that were occupied by cells in one sample. The following subsection discusses how the distribution of cells with respect to the tree can be used for classification.

### Classification Based on One Tube

The above SPADE analysis derived 359 distributions: how cells in tube 2 of each sample were distributed across the tree. Such a distribution is a characteristic of each sample that can be used for classification. One possible way to address the classification challenge is to ask: whether the cell percentage of any subtree correlates with the normal/AML phenotype. Since each subtree can be considered as one cell type or a collection of a few similar cell types, this analysis identifies cell types whose abundance predicts the normal/AML phenotype. For the tree shown in Figure 2, the total number of possible subtrees is greater than 24000. Therefore, this analysis is subject to multiple hypothesis testing.

Instead of searching for subtrees that predict the phenotype, an alternative is to ask whether the entire distribution is predictive. Following this idea, my solution for the AML prediction challenge was to combine two algorithms: the earth mover’s distance (EMD) [28] and a nearest-neighbor classifier named Relief [22].

**Figure 4:**
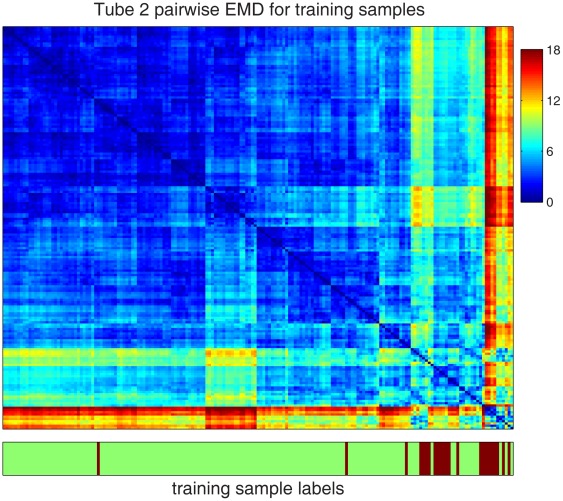
Pairwise earth mover’s distance (EMD) among all training samples, derived from tube 2. The values of EMD range from 0 to 18, as shown by the color bar. The bottom panel shows the class label of each training sample.

EMD is a distance metric that measures the dissimilarity between two probability distributions with respect to a structured domain [28], which is the SPADE tree in this analysis. If one unit of effort is needed to move one cell from a tree node to its adjacent neighbor, the EMD between two distributions in Figure 3 is the minimum effort needed to make one distribution the same as the other by moving cells. It can be calculated by solving a constraint linear programming problem. Based on the data and the tree derived from tube 2, the pairwise EMDs of all training samples were calculated and shown in the heatmap in Figure 4. The order of the samples in the heatmap was organized by hierarchical clustering, so that similarity patterns among the samples was visible along the diagonal line [31]. The normal and AML samples were not perfectly separated according to the EMD values. However, the AML samples formed more than two clusters in the bottom-right corner of Figure 4, indicating that the AML samples can be further divided into a few subtypes according to the markers measured in tube 2.

**Figure 5:**
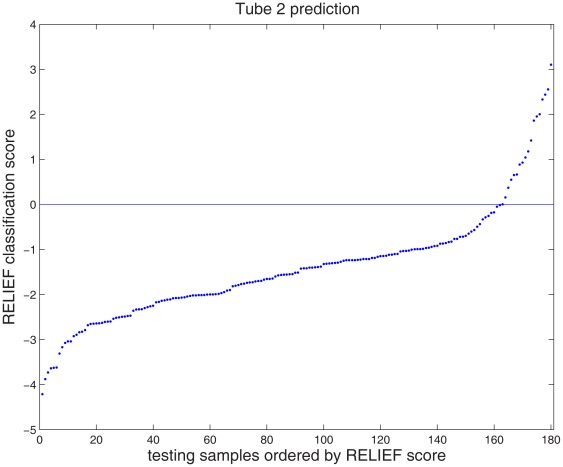
Relief score for each testing sample, derived from tube 2. To make predictions, scores should be compared with a threshold of 0. A positive score means the testing sample is likely to be AML; whereas a negative score means normal.

Relief is a nearest neighbor based classifier. The Relief score for one testing sample is defined by the distance from the testing sample to the nearest normal sample minus the distance from the test sample to the nearest AML sample. If a testing sample is normal, the distance between it and the nearest normal sample is likely to be small, and the distance between it and the nearest AML sample is likely to be large. Thus, the score for a normal testing sample is likely to be negative. Following similar logic, the score for an AML sample is likely to be positive. Therefore, the phenotype of a testing sample was predicted by comparing its score against 0. Using the EMDs derived from the data of tube 2, the scores for the 180 testing samples were computed and shown in Figure 5, where the samples were ordered by sorting their scores. Based on the data of tube 2, this approach predicted that 18 testing samples were AML. The Relief scores of three samples (one predicted as AML and two predicted as normal) were quite close to the threshold 0, indicating that the predictions for those three samples were of low confidence.

### Classification Based on All Tubes

**Figure 6:**
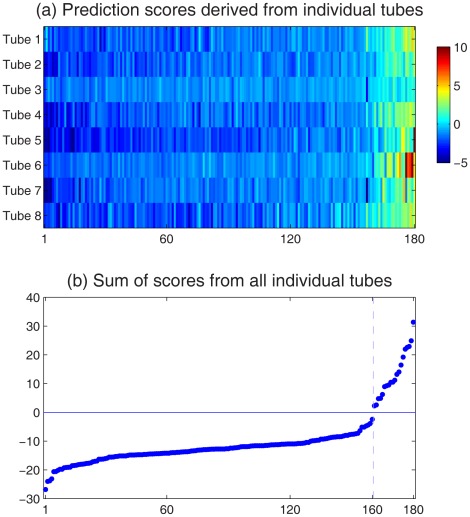
Final prediction scores. (a) The Relief prediction scores for testing samples, derived from individual tubes. Each row corresponds to one tube. Along the horizontal axis, the 180 testing samples are ordered by sorting the sum of the scores from all 8 tubes. (b) Sum of scores from all 8 tubes. Samples are in the same order as above. The final prediction is made by comparing the scores against the threshold 0. A clear gap can be observed around 0, indicating high confidence of the prediction.

The EMD and Relief analysis for tube 2 was performed on all the individual tubes (results available in information S2). Each tube provided a set of scores for the 180 testing samples. The 8 sets of scores are shown in Figure 6(a), in which the testing samples were ordered by sorting the sum of the scores from all 8 tubes. The 8 sets of scores appeared to be highly correlated, suggesting that different tubes produced similar prediction results. The sum of the 8 sets of scores is shown in Figure 6(b), where the samples were in the same order as Figure 6(a). The final prediction was made by comparing the summed scores against 0. The summed scores of 20 testing samples were positive and were predicted to be AML. The clear gap around 0 indicates that the prediction was of high confidence. This prediction was submitted to DREAM6 before the gold standard was released. After the DREAM6 challenge ended, I was notified that the prediction result was 100% accurate.

## Discussion

This paper describes a novel framework for predicting phenotypic properties from single-cell data. The framework contains three main components: SPADE, EMD and Relief. The role of SPADE is to perform feature extraction. SPADE clusters cells and constructs a tree that captures the relationship among the cell clusters. Such a tree representation can be used to summarize the single-cell data for each sample into a distribution of cells with respect to the tree. The cell distribution is one feature extracted from the data. EMD is a distance metric suitable for comparing the cell distributions in different samples, while taking the tree into account. The EMD between all pairs of samples forms a kernel matrix that can be fed into any classifiers in the machine learning literature. The classifier used in this paper is Relief, which is a nearest neighbor based approach.

Using the same framework, one can construct other pipelines for predicting phenotypes from single-cell data. For example, the SPADE feature extraction component can be replaced by manual gating [5], [6] or clustering algorithms [7]–[15]; the EMD metric can be replaced by the Euclidean distance; and the Relief classifier can be replaced by support vector machine [18] or other classifiers. Some of those possible pipelines may achieve similar prediction performance as that described in this paper. For example, a few other participating teams in the DREAM6 AML prediction challenge also achieved 100% accuracy. The major difference between those possible pipelines and that in this paper is the topology of the SPADE tree, which aims to capture the relationship among subpopulations of cells.

The proposed framework handles the individual tubes separately. When the results from the individual tubes were combined to form a final prediction, the different tubes were considered equally. The reason was that the predictions from the individual tubes were highly similar, as shown in Figure 6(a). Details of the prediction performance based on the individual tubes are available in information S3. Even the isotype and unstained controls (tubes 1 and 8) were able to produce predictions that had reasonably high accuracy. If the final scores were defined as the sum of tubes 2–7, the prediction result would have been identical to that based on all tubes. Combining the different tubes with equal weight may not be optimal. However, such an approach is sufficient for the AML prediction dataset.

## Methods

### SPADE

Spanning-tree progression analysis of density-normalized events (SPADE) contains four computational components: density-dependent downsampling, agglomerative clustering, minimum-spanning tree construction, and upsampling. Details are available in Qiu et al [16]. To make this paper more self-contained, brief descriptions of the algorithm and parameter settings are provided.

The downsampling component throws away cells in a density-dependent manner. SPADE keeps cells using the following probability:
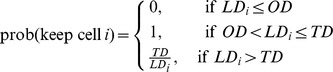
where 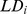 is the local density for cell *i*, which is the number of cells within its neighborhood. The neighborhood size is 5 times of the median L1 distance from a randomly chosen cell to its nearest neighbor. 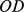 is the outlier density, defined as the 1st percentile of the local densities of all cells. 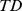 is the target density, chosen such that 2000 cells will survive the downsampling process. This was performed separately for each tube of each sample.

After downsampling, cells in the same tube of all 359 samples were pooled, resulting in a set of 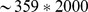 cells. Since the number of cells after pooling was too large for the subsequent clustering step, the pooled downsampled data was further uniformly downsampled to 50000 cells, a size that was within the capacity of the clustering component of SPADE.

The clustering component of SPADE is a variation of the agglomerative hierarchical clustering algorithm. SPADE clustering encourages different clusters to have similar sizes, so that the resulting clusters are relatively balanced compared to those produced by standard hierarchical clustering. The stopping criterion of the agglomerative process is a user-defined desired number of clusters, which was set at 150.

After clustering, each cluster is represented by its median expression of the measured markers, and the distance between each pair of clusters is defined by the L1 distance. SPADE constructs a minimum spanning tree that links the cell clusters with minimum total edge length, using the Boruvka’s algorithm [32]. When visualizing the tree, a modification of the Fruchterman and Reingold algorithm [16], [33] is used to automatically determine a layout. Such a layout faithfully reflects the topology of the tree. However, the edge length information of the tree is not encoded in the visualization.

Finally, upsampling is performed to recover the information lost during the downsampling process. For each cell in one tube of each sample, SPADE identifies its nearest neighbor in the subset of 50000 cells used in clustering, and assigns it to the cluster that its nearest neighbor belongs to. After upsampling, each cell in the original dataset is assigned to one cluster/node. The median marker expression and the cell count of each node can be calculated based on the entire original dataset.

The SPADE analysis in this paper was performed using SPADE2.0, an efficient Matlab implementation of the algorithm. SPADE2.0 is about 15 times faster than the original prototype released when SPADE was first published [16], and includes an easy-to-use graphical user interface. SPADE2.0 is available at http://odin.mdacc.tmc.edu/pqiu/software/SPADE2/index.html.

### Earth Mover’s Distance

The earth mover’s distance (EMD) measures the distance between two probability distributions [28]. In this work, EMD is used to evaluate the distance between two cell distributions with respect to a tree structure (see examples in Figure 3). Imagine the cells in one node as the mass in one city, the tree edges as the highways that connect different cities, and efforts are needed to move mass from city to city along the highways. The EMD between two distributions is the minimum amount of effort needed to make one distribution the same as the other. Here, the cost for moving one cell from a tree node to its adjacent neighbor is defined as one unit of effort. The EMD between two cell distributions (*P* and *Q*) can be obtained by a linear programming formulation:
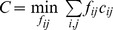


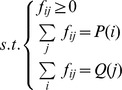



To make 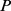 the same as 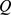, 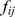 is the number of cells moved from node 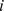 to node 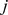, and 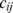 is the number of hops in the shortest path between the two nodes. The equality constraints ensure that the total number of cells moved out of node 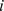 equals 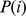, and the total number of cells moved into node 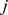 equals 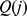. The solution to this minimization problem is the EMD between the two distributions, and can be obtained by the “linprog” function in Matlab or other linear programming solvers. One possible extension of this formulation is to include the edge length information in the cost matrix, defining 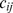 as the total edge length of the shortest path connecting the two nodes.

## Supporting Information

Information S1 **Data quality check figures.**
(PDF)

Information S2 **EMD and RELIEF figures based on individual tubes.**
(PDF)

Information S3 **Classification based on individual or subsets of tubes.**
(PDF)
