# Supplementary material for: Inferring Phenotypic Properties from Single-Cell Characteristics
Source: PLoS One. 2012 May 25;7(5):e37038. doi: 10.1371/journal.pone.0037038 (PMC3360688; doi:10.1371/journal.pone.0037038)
Supplement: Information S1 — Data quality check figures. (PDF) [file pone.0037038.s001.pdf]

S1. Data quality check figures

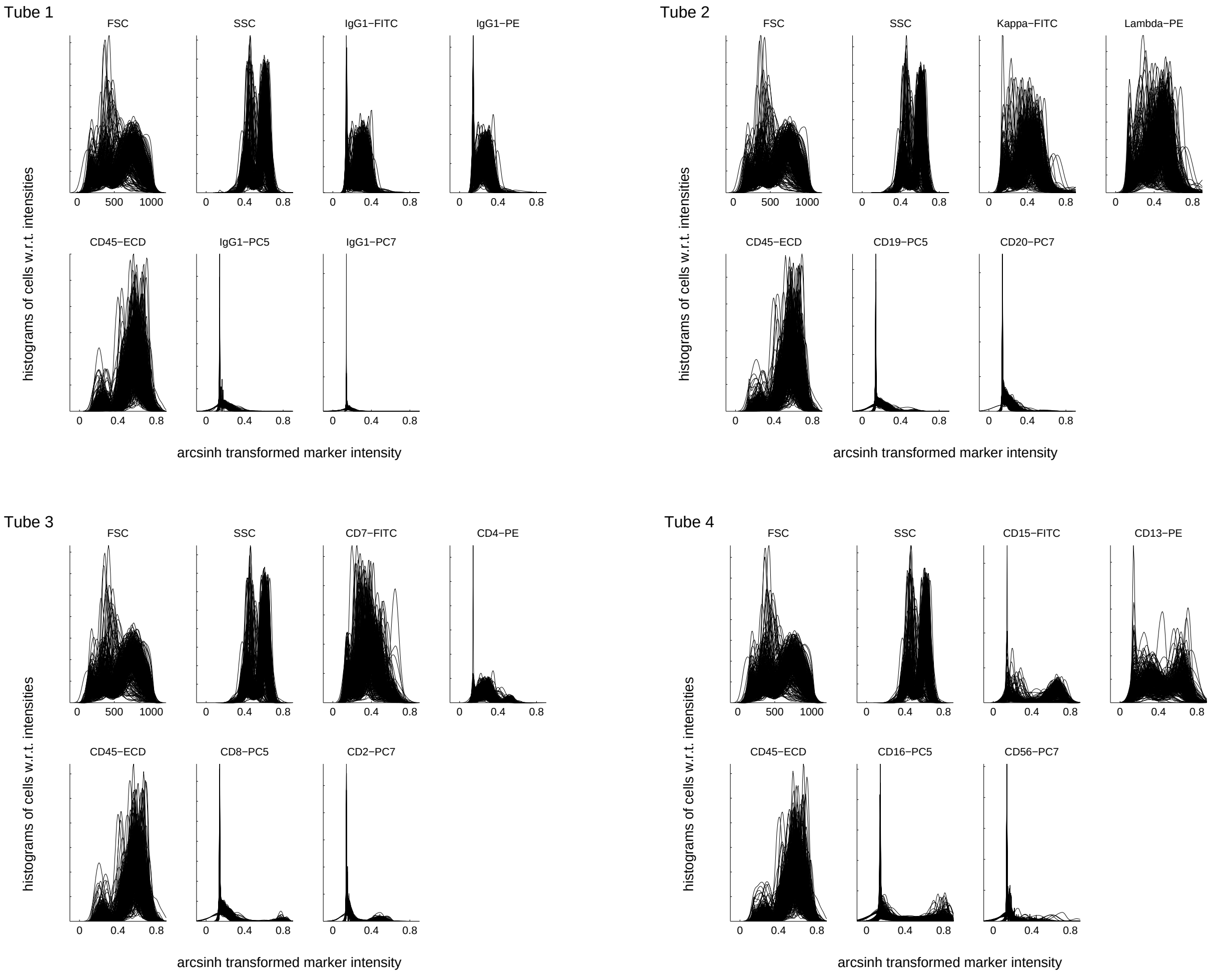

Figure S1: Distributions of marker intensities in data of tubes 1-4. Each small panel contains 359 curves, and each curve shows the distributions of the intensities of one marker in one tube for one of the 359 samples.

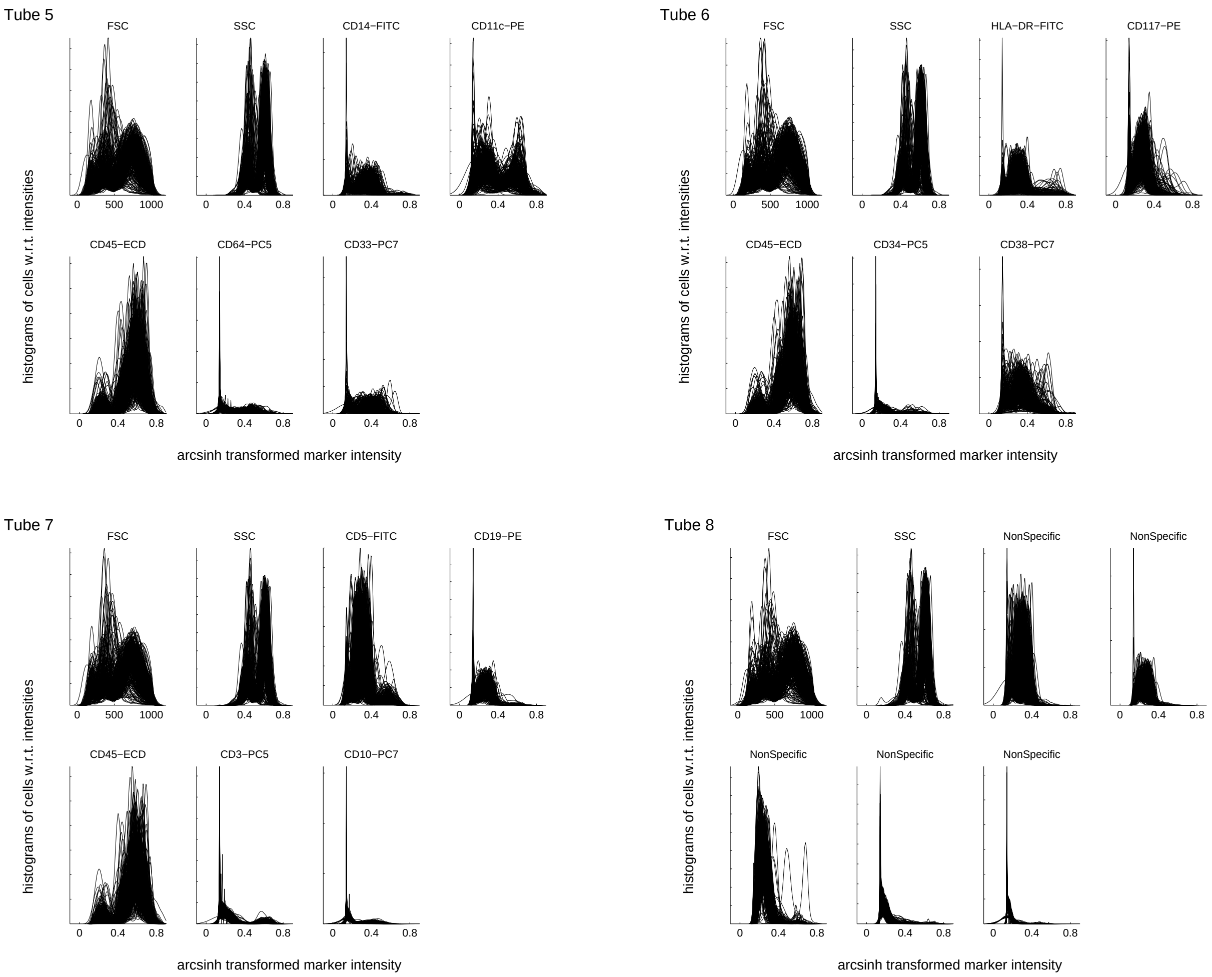

Figure S2: Distributions of marker intensities in data of tubes 5-8. Each small panel contains 359 curves, and each curve shows the distributions of the intensities of one marker in one tube for one of the 359 samples.
