# Supplementary material for: Inferring Phenotypic Properties from Single-Cell Characteristics
Source: PLoS One. 2012 May 25;7(5):e37038. doi: 10.1371/journal.pone.0037038 (PMC3360688; doi:10.1371/journal.pone.0037038)
Supplement: Information S2 — EMD and RELIEF figures based on individual tubes. (PDF) [file pone.0037038.s002.pdf]

S2. EMD and RELIEF figures based on individual tubes

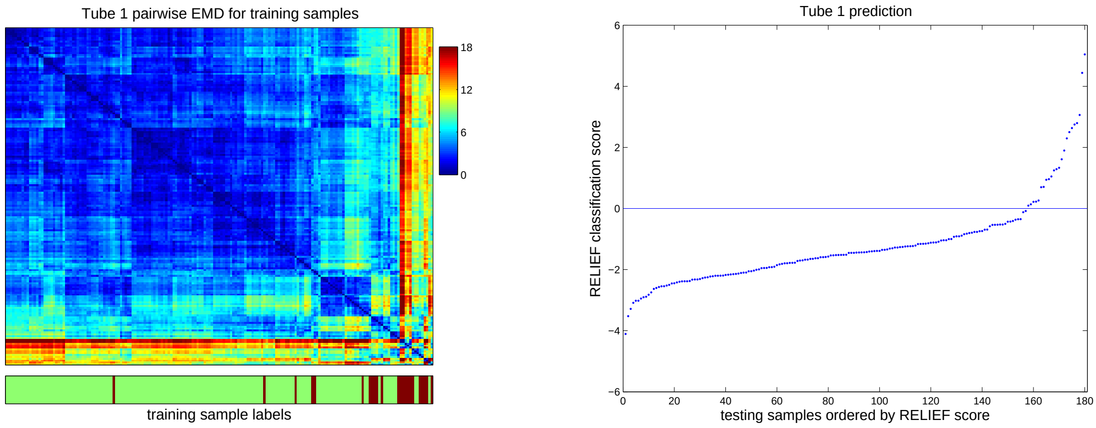

Figure S3: EMD and RELIEF analysis of tube 1. (a) Pairwise EMD distance for all training samples. (b) RELIEF scores for testing samples.

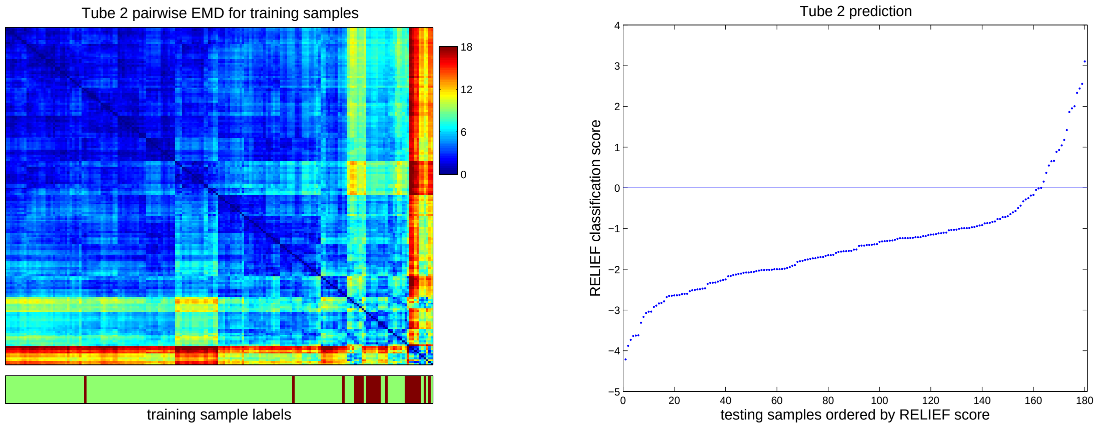

Figure S4: EMD and RELIEF analysis of tube 2. (a) Pairwise EMD distance for all training samples. (b) RELIEF scores for testing samples.

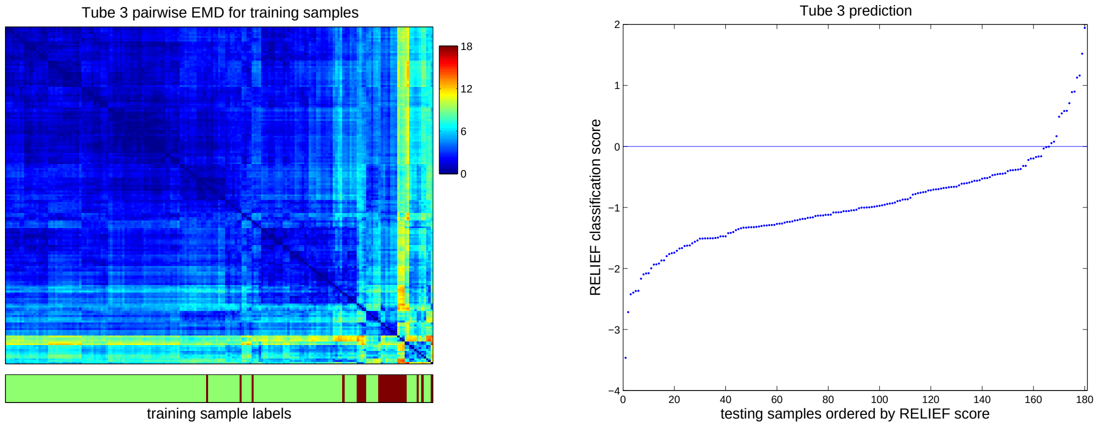

Figure S5: EMD and RELIEF analysis of tube 3. (a) Pairwise EMD distance for all training samples. (b) RELIEF scores for testing samples.

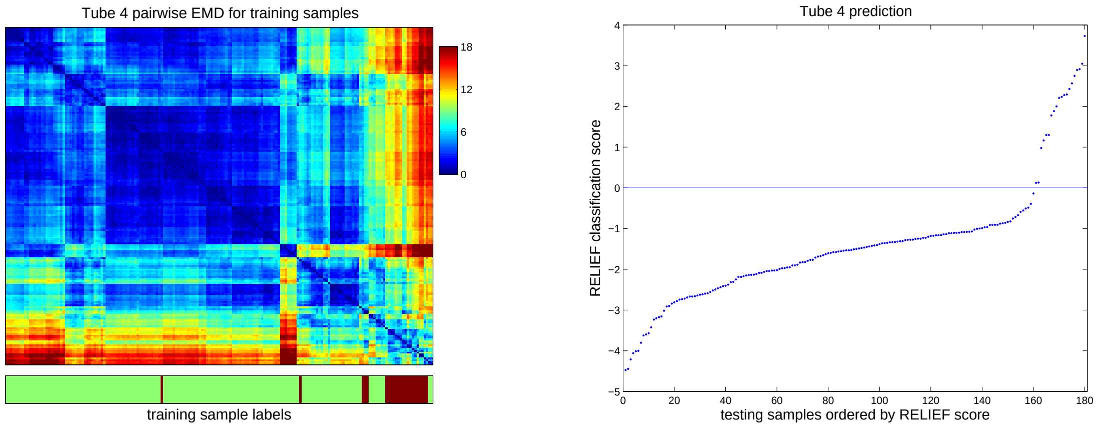

Figure S6: EMD and RELIEF analysis of tube 4. (a) Pairwise EMD distance for all training samples. (b) RELIEF scores for testing samples.

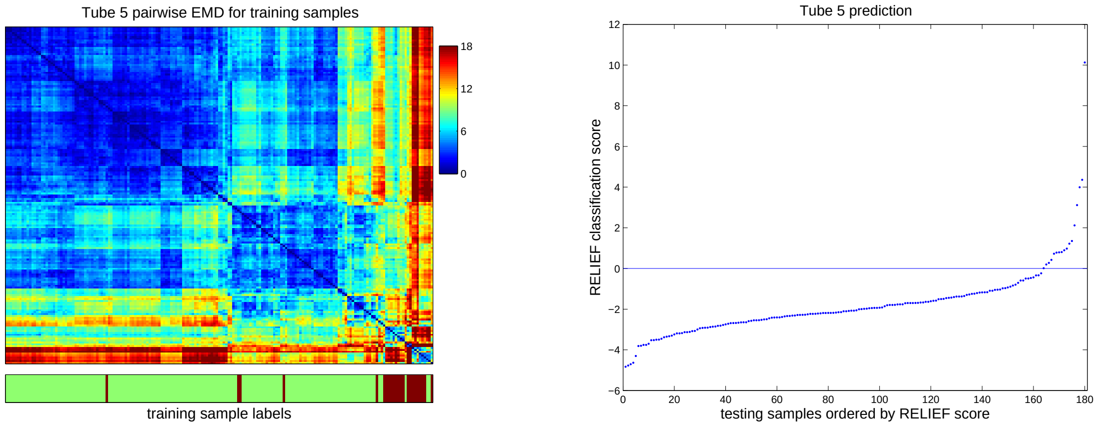

Figure S7: EMD and RELIEF analysis of tube 5. (a) Pairwise EMD distance for all training samples. (b) RELIEF scores for testing samples.

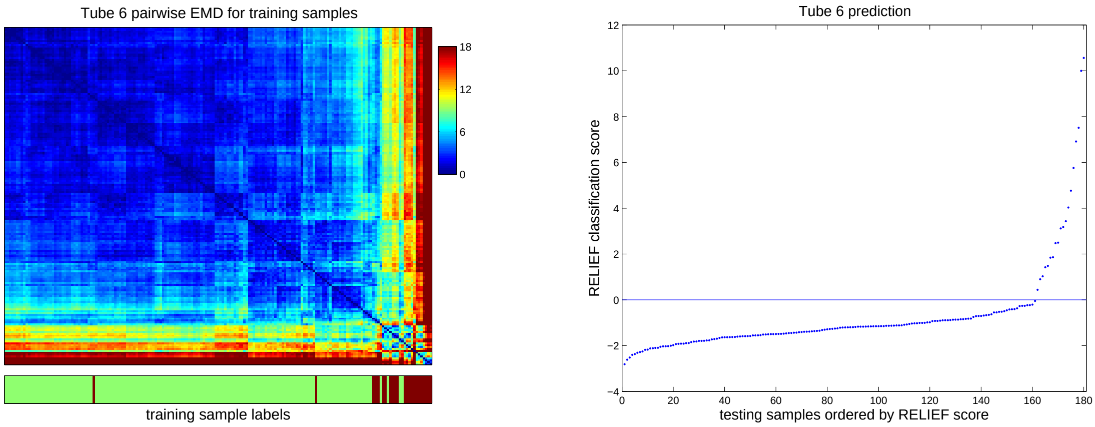

Figure S8: EMD and RELIEF analysis of tube 6. (a) Pairwise EMD distance for all training samples. (b) RELIEF scores for testing samples.

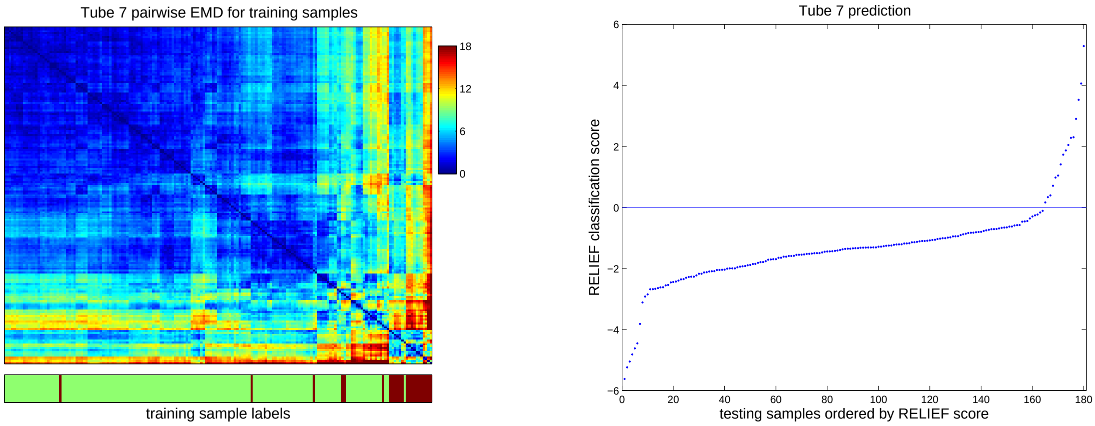

Figure S9: EMD and RELIEF analysis of tube 7. (a) Pairwise EMD distance for all training samples. (b) RELIEF scores for testing samples.

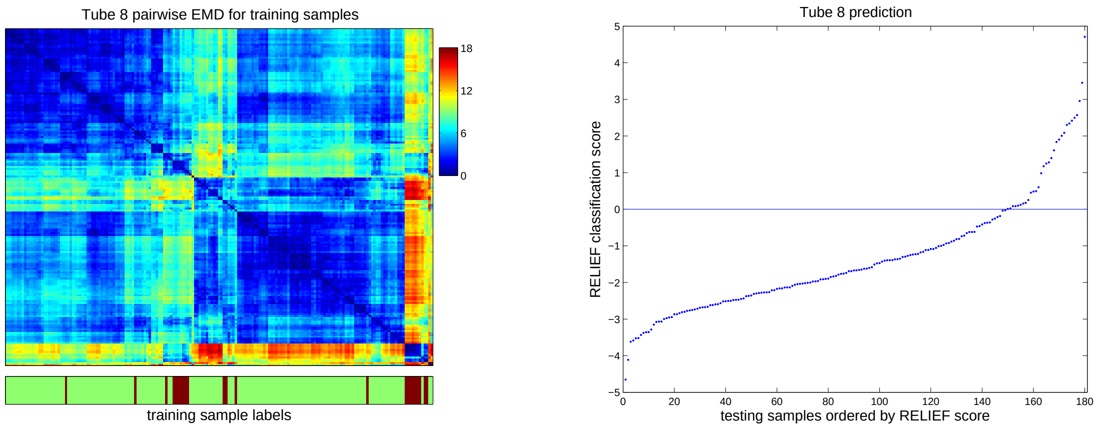

Figure S10: EMD and RELIEF analysis of tube 8. (a) Pairwise EMD distance for all training samples. (b) RELIEF scores for testing samples.
