# Supplementary material for: Inferring Phenotypic Properties from Single-Cell Characteristics
Source: PLoS One. 2012 May 25;7(5):e37038. doi: 10.1371/journal.pone.0037038 (PMC3360688; doi:10.1371/journal.pone.0037038)
Supplement: Information S3 — Classification based on individual or subsets of tubes. (PDF) [file pone.0037038.s003.pdf]

S3. Classification based on individual or subsets of tubes

Table S1: Prediction results obtained by either SPADE+EMD+RELIEF applied to individual tubes, or summary using subsets of the tubes.

	number of predicted normal	number of predicted AML	Precision	Recall	prediction scores
Tube 1 - isotype	157	23	0.78	0.90	Figure S3
Tube 2	162	18	1	0.90	Figure S4
Tube 3	166	14	1	0.70	Figure S5
Tube 4	160	20	0.90	0.90	Figure S6
Tube 5	163	17	0.94	0.80	Figure S7
Tube 6	161	19	0.95	0.90	Figure S8
Tube 7	164	16	1	0.80	Figure S9
Tube 8 - unstained	149	31	0.61	0.95	Figure S10
Tubes 2-7	160	20	1	1	not shown
Tubes 1-8	160	20	1	1	Figure 6
